# Magnesium Isoglycyrrhizinate Reduces Hepatic Lipotoxicity through Regulating Metabolic Abnormalities

**DOI:** 10.3390/ijms22115884

**Published:** 2021-05-30

**Authors:** Li Lu, Kun Hao, Yu Hong, Jie Liu, Jinwei Zhu, Wenjiao Jiang, Zheying Zhu, Guangji Wang, Ying Peng

**Affiliations:** 1 Key Lab of Drug Metabolism and Pharmacokinetics, State Key Laboratory of Natural Medicines, China Pharmaceutical University, 24 Tong Jia Xiang, Nanjing 210009, China; Li.Lu@nottingham.ac.uk (L.L.); haokun@cpu.edu.cn (K.H.); hesy0430@outlook.com (Y.H.); 18851103858@163.com (J.L.); Zhujw2015cpu@163.com (J.Z.); wenjiaojiangcpu@126.com (W.J.); 2 Division of Molecular Therapeutics & Formulation, School of Pharmacy, University Park Campus, The University of Nottingham, Nottingham NG7 2RD, UK; pazzl1@exmail.nottingham.ac.uk

**Keywords:** magnesium isoglycyrrhizinate, palmitic acid, hepatic lipotoxicity, metabonomics, lipidomics

## Abstract

The excessive accumulation of lipids in hepatocytes induces a type of cytotoxicity called hepatic lipotoxicity, which is a fundamental contributor to liver metabolic diseases (such as NAFLD). Magnesium isoglycyrrhizinate (MGIG), a magnesium salt of the stereoisomer of natural glycyrrhizic acid, is widely used as a safe and effective liver protectant. However, the mechanism by which MGIG protects against NAFLD remains unknown. Based on the significant correlation between NAFLD and the reprogramming of liver metabolism, we aimed to explore the beneficial effects of MGIG from a metabolic viewpoint in this paper. We treated HepaRG cells with palmitic acid (PA, a saturated fatty acid of C16:0) to induce lipotoxicity and then evaluated the antagonistic effect of MGIG on lipotoxicity by investigating the cell survival rate, DNA proliferation rate, organelle damage, and endoplasmic reticulum stress (ERS). Metabolomics, lipidomics, and isotope tracing were used to investigate changes in the metabolite profile, lipid profile, and lipid flux in HepaRG cells under different intervention conditions. The results showed that MGIG can indeed protect hepatocytes against PA-induced cytotoxicity and ERS. In response to the metabolic abnormality of lipotoxicity, MGIG curtailed the metabolic activation of lipids induced by PA. The content of total lipids and saturated lipids containing C16:0 chains increased significantly after PA stimulation and then decreased significantly or even returned to normal levels after MGIG intervention. Lipidomic data show that glycerides and glycerophospholipids were the two most affected lipids. For excessive lipid accumulation in hepatocytes, MGIG can downregulate the expression of the metabolic enzymes (GPATs and DAGTs) involved in triglyceride biosynthesis. In conclusion, MGIG has a positive regulatory effect on the metabolic disorders that occur in hepatocytes under lipotoxicity, and the main mechanisms of this effect are in lipid metabolism, including reducing the total lipid content, reducing lipid saturation, inhibiting glyceride and glycerophospholipid metabolism, and downregulating the expression of metabolic enzymes in lipid synthesis.

## 1. Introduction

In order to proliferate and survive, cells need to continuously obtain fatty acids (FFAs) to provide energy, support membrane synthesis, and participate in cellular signaling [1]. Cells can obtain FFAs through external uptake or de novo synthesis and then use these FFAs to synthesize lipids, such as triglycerides and glycerophospholipids [2]. Triglycerides are the main means of storage and transportation of FFAs in cells [3] and are also the most abundant lipids in the body; most tissues can use the decomposition products of triglycerides to provide energy [4]. Glycerophospholipids, the most abundant phospholipids in the body, are not only some of the main lipid components of cell membranes, but also participate in recognition and signal transduction on cell membranes [4,5]. However, the excessive accumulation of lipids and their intermediate products in nonadipose tissues causes metabolic abnormalities and cell death in a process known as lipotoxicity [6]. Lipotoxicity due to lipid accumulation is a metabolic syndrome that can cause various diseases, including insulin resistance, heart failure, and liver steatosis [7]. Hepatic lipotoxicity occurs when the capacity of hepatocytes to manage and export FFAs is overwhelmed, leading to hepatocyte dysfunction or hepatocyte death/apoptosis [8]. In nonalcoholic fatty liver disease (NAFLD), the lipotoxic apoptosis of hepatocytes is an important initial event in the diagnosis of the disease, and this phenomenon continues to occur during the development of the disease [9].

NAFLD is one of the most common liver diseases worldwide, and is marked by hepatic fat accumulation that is not due to alcohol abuse [10,11]. There are no effective interventions for NAFLD. Magnesium isoglycyrrhizinate (MGIG), the magnesium salt of a stereoisomer of natural glycyrrhizic acid, is widely used as a hepatocyte protective agent due to its potent anti-inflammatory and hepatoprotective activities [12,13,14,15]. Some studies have illustrated that MGIG has protective effects against NAFLD, including the prevention of lipid-accumulation-induced cell apoptosis [16,17]. However, the specific mechanism of action remains unknown. NAFLD is considered to be highly associated with a thorough reprogramming of hepatic metabolism [18]; therefore, we sought here to elucidate the antagonistic effects of MGIG on hepatic lipotoxicity from the perspective of the endogenous metabolism of hepatocytes. Metabolomics is a powerful tool for large-scale studies of endogenous metabolites (including sugars, lipids, amino acids, nucleic acids, organic acids, carbohydrates, etc.) [19]. Unlike other omics methods, metabolomics directly reflects the current cell state and biochemical activity [20,21]. Lipidomics is a branch of metabolomics. Lipids account for a large proportion of all endogenous metabolites (for example, about 70% of the metabolites in plasma are lipids), and lipids play important roles in a wide range of biological processes [22,23]. Therefore, this article aimed to investigate the impact of the regulation of MGIG on the endogenous metabolism of hepatocytes under palmitic-acid-induced lipotoxicity through the fingerprints of the metabolome and lipidome in hepatocytes, obtained using metabolomics and lipidomics techniques. Furthermore, we explored the relevant molecular mechanism of MGIG’s protective effect on hepatocytes from the perspective of metabolism.

## 2. Results

### 2.1. The Protective Effect of MGIG on PA-INDUCED Cytotoxicity

**Figure 1:**
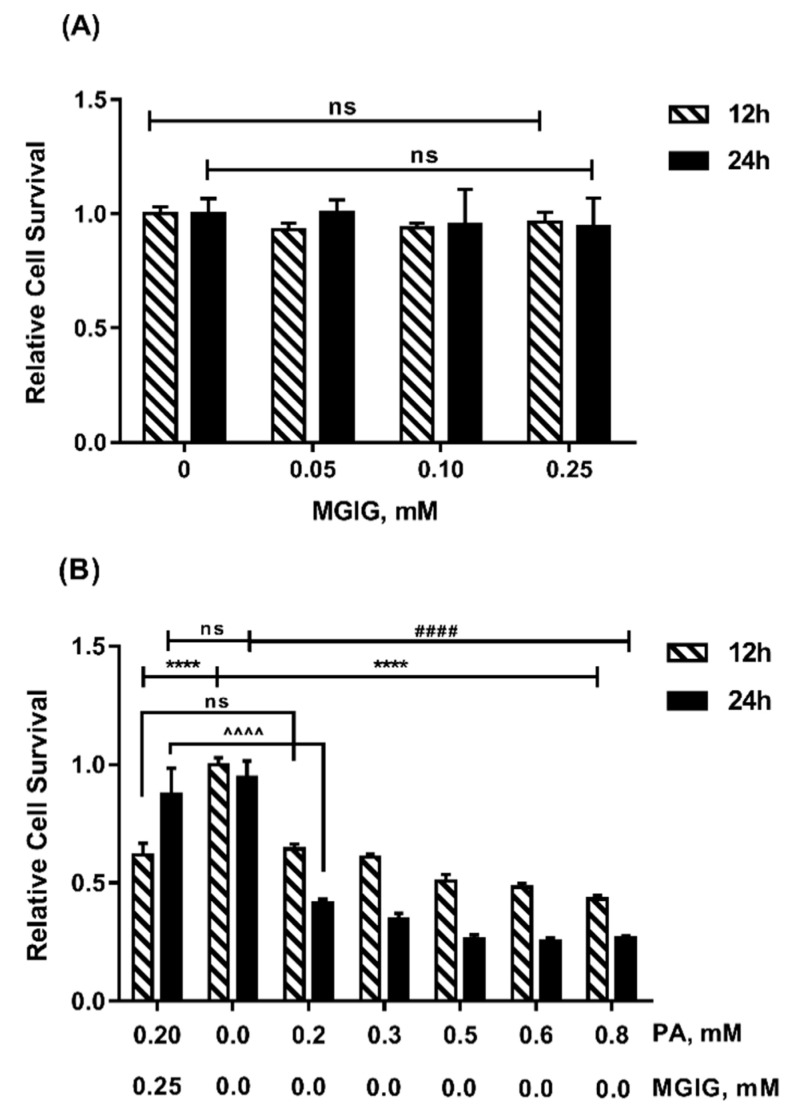
The effect of palmitic acid (PA) and magnesium isoglycyrrhizinate (MGIG) on the proliferation of HepaRG cells after 12 h or 24 h incubation, evaluated via CCK-8. (**A**) Relative cell survival of HepaRG cells after administration of different concentrations (0, 0.05, 0.10, 0.25 mM) of MGIG; (**B**) Relative cell survival of HepaRG cells after administration of different concentrations (0, 0.2, 0.3, 0.5, 0.6, 0.8 mM) of PA alone and 0.2 mM PA plus 0.25 mM MGIG. Data are expressed as the mean ± SD (*n* = 6). For the statistical analysis between the treated group (PA alone or MGIG or the two together) and the control group (no PA and MGIG) at 12 h and 24 h, respectively, “ns” means *p* > 0.05, **** means *p* < 0.0001 (12 h), and ^####^ means *p* < 0.0001 (24 h). For the statistical analysis between PA alone group and PA plus MGIG combined group, “ns” means *p* > 0.05, ^^^^^^ means *p* < 0.0001.

Palmitic acid (PA, C16:0) is one of the most common long-chain saturated fatty acids in food and in the human body. Too much palmitic acid can cause cells to undergo mitochondrial oxidative stress, endoplasmic reticulum stress, cellular inflammation, and ultimately cell death [24]. As shown in Figire 1B, PA significantly inhibited the proliferation of HepaRG cells in a concentration-dependent and time-dependent manner. At the lowest tested concentration (0.2 mM) of PA, the relative survival rates of HepaRG cells were only 64% and 42% after the administration of PA for 12 h and 24 h, respectively, which was significantly different from that of untreated cells (*p* < 0.0001). However, MGIG had no effect on the proliferation of HepaRG cells (Figure 1A). When PA (0.2 mM) was administered plus MGIG (0.25 mM), the relative cell survival rates after 12 h and 24 h incubation were 62% and 87%, respectively. Compared with the PA-alone group, the cell survival rate of the MGIG intervention group after 24 h of incubation was significantly improved (*p* < 0.0001), being almost equivalent to that of the untreated cells. The results showed that MGIG had a protective effect against PA-induced cytotoxicity in HepaRG cells. In subsequent experiments, 0.2 mM PA stimulation for 24 h was used to establish the lipotoxicity model of HepaRG cells, and 0.25 mM MGIG was selected as the drug intervention concentration.

**Figure 2:**
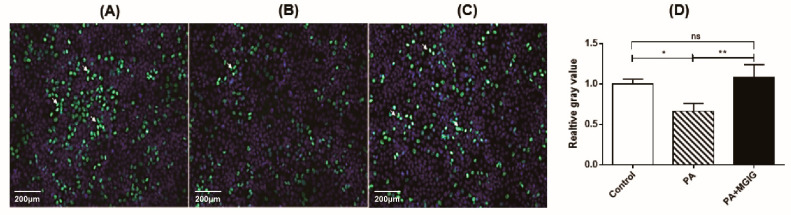
The effect of palmitic acid (PA) and magnesium isoglycyrrhizinate (MGIG) on the proliferation of HepaRG cells, evaluated via Edu488. (**A**) Typical image of control cells without drug treatment; (**B**) Typical image of cells after administration of 0.2 mM PA for 24 h; (**C**) Typical image of cells after administration of 0.2 mM PA plus 0.25 mM MGIG for 24 h; (**D**) The fluorescence intensity of Edu488 in the three tested groups (mean ± SD, *n* = 3). In the image, the scale bar is 200 μm, the blue is the nucleus (Hoechst 33342 staining), and the green is the newly synthesized DNA strand (EdU488 staining). One-way ANOVA analysis was applied for data comparison between groups; “ns” means *p* > 0.05, * means *p* < 0.05, and ** means *p* < 0.01.

The protective effect of MGIG for cell viability was verified not only by the overall proliferation rate, but also by the DNA synthesis rate. The newly synthesized DNA was labeled using the EdU method [25] and emitted green fluorescence. As shown in Figure 2, the green fluorescence significantly decreased in the PA-alone group. However, under the intervention of MGIG, the fluorescence significantly increased and returned to the level of the control group. 

### 2.2. The Protective Effect of MGIG against PA-Induced Organelle Damage

**Figure 3:**
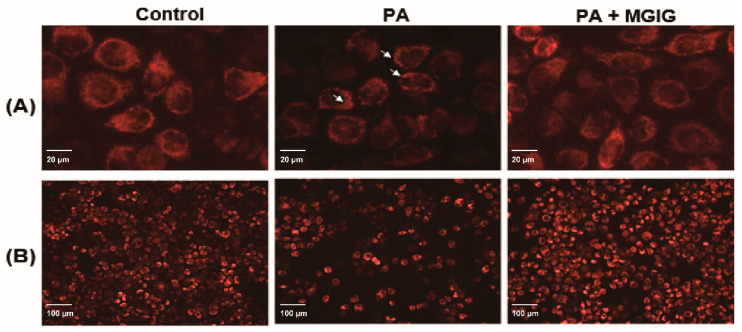
The effect of palmitic acid (PA) and magnesium isoglycyrrhizinate (MGIG) on the morphological structures of the endoplasmic reticulum and mitochondria of HepaRG cells. (**A**) Typical image of the endoplasmic reticulum stained with the ER tracker (red), the scale bar is 20 μm. (**B**) Typical image of mitochondria stained with TMRM (red), the scale bar is 100 μm. The control group was untreated cells; the PA group was cells administered 0.2 mM PA for 24 h; the PA + MGIG group was cells administered 0.2 mM PA plus 0.25 mM MGIG for 24 h. *n* = 3 in each group. Arrow: the fragmented ice floe structures of the endoplasmic reticulum membrane.

We further studied the regulation effects of MGIG on mitochondrial stress and endoplasmic reticulum (ER) stress caused by lipotoxicity. When the organelle undergoes a stress response, its corresponding morphological structure will also change. Therefore, we used ER-Tracker Red staining and TMRM staining to observe the morphological structures of the ER and mitochondria, respectively. The ER is the largest membrane system and the main site of lipid synthesis in cells. Yihui Shen et al. found that PA can induce the ER membrane to present fragmented ice floe structures, resulting in loss of the membrane integrity and causing the fragmented ER membrane to gather around the nucleus [26]. As shown in Figure 3A, the ER membrane in the HepaRG cells did show obvious morphological changes after PA administration, such as fragmented ice floe structures, but the ER membrane was able to maintain good integrity after MGIG intervention. The oxidation of fatty acids in the mitochondria causes the electron-transport respiratory chain to produce a certain amount of ROS. However, excessive ROS cause damage to the structure and function of the mitochondria. TMRM dye can track the inner membrane of mitochondria, and the intensity of fluorescence reflects their vitality. As shown in Figure 3B, the red fluorescence was significantly reduced after PA administration, and the fluorescence intensity was significantly increased after MGIG intervention. The results showed that MGIG had protective effects against damage to the mitochondria and ER in the PA-induced lipotoxicity model.

### 2.3. The Regulatory Effect of MGIG on PA-Induced Metabolic Reprogramming

**Figure 4:**
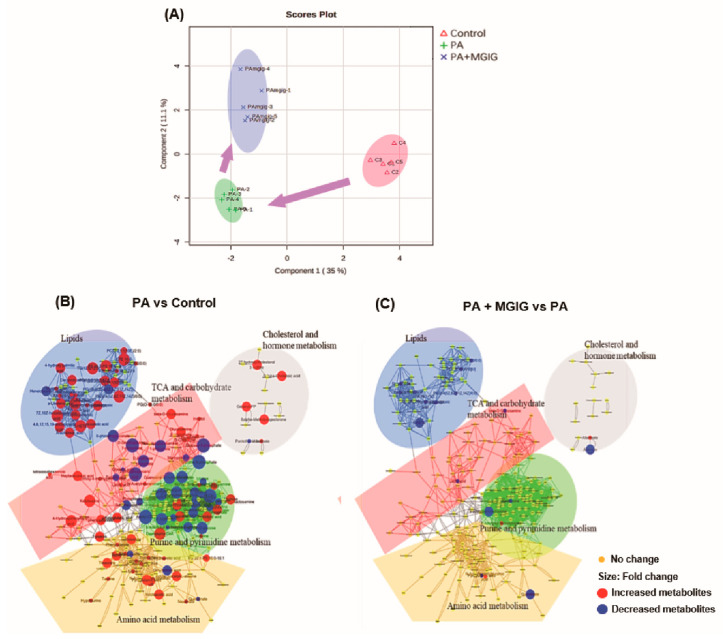
The regulatory effect of magnesium isoglycyrrhizinate (MGIG) on palmitic acid (PA)-induced metabolic reprogramming of HepaRG cells. (**A**) PLS-DA score plot for multivariate analysis of the three test groups; (**B**) Visual plot for all identified endogenous metabolites of the Control group and the PA group created using Cytoscape; (**C**) Visual plot for the PA group and the PA + MGIG group. The Control group was untreated cells; the PA group was cells administered 0.2 mM PA for 24 h; and the PA + MGIG group was cells administered 0.2 mM PA plus 0.25 mM MGIG for 24 h. *n* = 5 in each group.

Abnormal metabolism is an important feature of many stressed cells, and lipotoxicity can cause multiple stress responses in cells. Therefore, we used nontarget metabolomics to study PA-induced endogenous metabolic changes in HepaRG cells and the possible regulation of metabolic abnormalities by MGIG. First, through an sPLS-DA score plot of multivariate analysis (Figure 4A), we found the samples in the untreated group and the PA-induced lipotoxicity model group were clearly distinguished, indicating that the endogenous metabolites in HepaRG cells changed significantly after PA administration. A total of 116 metabolites were identified as differential metabolites, of which 76 metabolites in the model group were significantly upregulated and 40 metabolites were significantly downregulated. However, the samples from the MGIG intervention group also had a good discrimination from the samples of the model group, and there was a tendency to migrate to the control group. This phenomenon indicates that MGIG had a regulatory effect on endogenous metabolism under lipotoxicity, causing it to gradually return to a normal state. In addition, 25 metabolites were identified as differential metabolites, of which 20 were significantly downregulated after MGIG intervention and five were upregulated.

We used Cytoscape to visually display the metabolic networks involved in these differential metabolites and the changing trends of the differential metabolites (Figure 4B,C). As shown in Figure 4B, in the PA-induced lipotoxicity model, the differential metabolites were mainly concentrated in five types of metabolic pathways, including lipid metabolism, cholesterol and hormone metabolism, amino-acid metabolism, purine and pyrimidine metabolism, and TCA and carbohydrate metabolism. The abundance of these discriminant metabolites involved in these metabolic pathways was compared between the tested groups (see Supplementary Figure S4). All results showed that after PA induction, these typical cholesterol metabolites, lipid metabolites and amino acid metabolites increased significantly, while nucleotide metabolites decreased significantly. This means that PA stimulation can increase the metabolism of lipids, cholesterol and amino acids in HepaRG cells but reduce the metabolism of nucleotides. The results show that PA can induce excessive accumulation of lipids in cells by activating lipid metabolism in HepaRG cells, and may cause DNA damage by inhibiting nucleotide metabolism. However, for these changes in the discriminant metabolites, MGIG inhibited the increase of lipid metabolites, but had no significant effect on the changes of other metabolites. The results showed that MGIG had a regulatory effect on endogenous lipid metabolism in the PA-induced hepatocyte lipotoxicity model.

### 2.4. The Regulatory Effect of MGIG on PA-Induced Abnormal Lipid Metabolism

**Figure 5:**
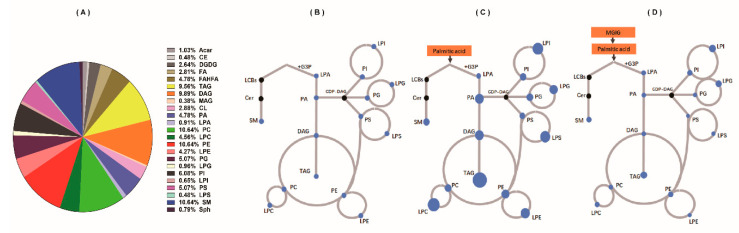
Lipid profile and lipid changes of HepaRG cells. (**A**) The annotated lipids found in the HepaRG cells using the MS-DIAL lipidomics database, including about 4000 lipids in 23 lipid subclasses; (**B**) Visual plot of the metabolic network of palmitic acid in the glycerophospholipid pathway in untreated cells, drawn using Cytoscape software (*n* = 5); (**C**) Visual plot of the changes in the content of differential lipids involved in the metabolic network of palmitic acid in cells administered 0.2 mM palmitic acid for 24 h (*n* = 5); (**D**) Visual plot of the content changes in cells administered 0.2 mM palmitic acid plus 0.25 mM magnesium isoglycyrrhizinate (MGIG) for 24 h (*n* = 5). Acar: Acyl Carnitine; CE: Cholesterol ester; DGDG: Digalactosyldiacylglycerol; FA: Free Fatty acids; FAHFA: Fatty acid esters of hydroxy fatty acids; TAG: Triglyceride; DAG: Diglyceride; MAG: Monoglycerides; CL: Bisphosphatidylglycerol; PA: Phosphatidic acid; LPA: Lysophosphatidic acid; PC: Phosphatidylcholine; LPC: Lysophosphatidylcholine; PE: phosphatidylethanolamine; LPE: Lysophosphatidylethanolamine; PG: Phosphatidylglycerol; LPG: Lysophosphatidylglycerol; PI: Phosphatidylinositol; LPI: Lysophosphatidylinositol; PS: Phosphatidylserine; LPS: Lysophosphatidylserine; SM: Sphingomyelin; Sph: Sphingosine.

**Figure 6:**
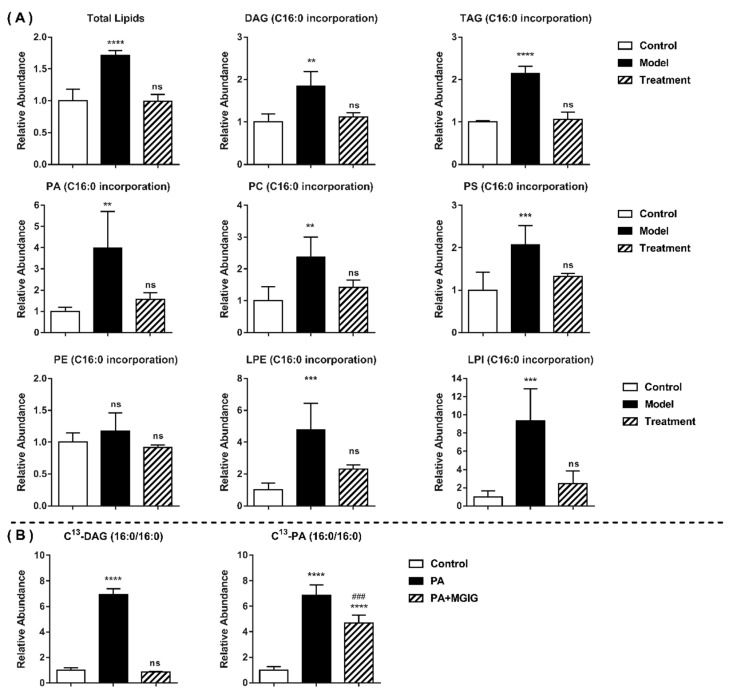
The content of lipids containing C16:0 (palmitic acid) components in HepaRG cells, detected using lipidomics. (**A**) Nonisotopically labeled palmitic acid was administered to induce lipid toxicity in HepaRG cells; (**B**) C13-isotope-labeled palmitic acid was administered to cells. The relative abundance of a class of lipids in the model group and the treatment group was obtained by normalizing the content in the control group. The Control group was untreated cells; the Model group was cells administered 0.2 mM palmitic acid for 24 h; the Treatment group was cells administered 0.2 mM palmitic acid plus 0.25 mM magnesium isoglycyrrhizinate for 24 h. Data are expressed as the mean ± SD (*n* = 5). In the statistical analysis carried out between the other groups and the control group, “ns” means *p* > 0.05, * *p* < 0.05; ** *p* < 0.01, *** *p* < 0.001; **** *p* < 0.0001. In the statistical analysis carried out between the Treatment group and Model group, ^###^ *p* < 0.001. DAG (C16:0 incorporation): the total content of a series of diglycerides containing C16:0; TAG: Triglyceride; PA: Phosphatidic acid; PC: Phosphatidylcholine; PS: Phosphatidylserine; PE: phosphatidylethanolamine; LPE: Lysophosphatidylethanolamine; LPI: Lysophosphatidylinositol. C^13^-DAG (16:0/16:0): one C^13^-isotope labelled diglyceride containing two molecules of C16:0; C^13^-PA (16:0/16:0): one C^13^-isotope labelled phosphatidic acid containing two molecules of C16:0.

We further applied lipidomic methods to investigate the changes in the lipid profile of HepaRG cells after palmitic-acid stimulation or MGIG intervention. Using the MS-DIAL lipidomics database, a total of more than 4000 lipid components in the studied HepaRG cells were annotated in the positive ion mode and the negative ion mode. Among them, fatty acids and fatty acid esters accounted for 7.6%, phospholipids accounted for 56.8%, glycerol lipids accounted for 19.8%, and sphingolipids accounted for 11.0% (Figure 5A). The glycerol lipids were mainly triglycerides and diglycerides. The phospholipids included bisphosphatidylglycerol, phosphatidic acid, phosphatidylcholine, phosphatidylethanolamine, phosphatidylglycerol, phosphatidylinositol, and phosphatidylserine. According to the lipidomic results, the glycerophospholipid pathway was the most abundant lipid metabolism pathway in the HepaRG cells under study. After administration of palmitic acid (C16:0), C16:0-CoA was first produced in the HepaRG cells, then this combined with 3-phosphoglycerol to form lysophosphatidic acid (16:0), before lysophosphatidic acid combined with another fatty acyl-CoA to form phosphatidic acid, and finally phosphatidic acid was used to synthesise other glycerides. We used Cytoscape to visually display this metabolic network of palmitic acid in the glycerophospholipid pathway (Figure 5B) and the changing trend of differential lipid metabolites after palmitic-acid induction and MGIG intervention (Figure 5C,D). The results showed that, after palmitic-acid stimulation, the content of most glycerides and glycerophospholipids in the HepaRG cells increased significantly. Compared with the control group, the total lipid content of the model group increased 1.7-fold (Figure 6A). However, after MGIG interfered with the palmitic-acid model, only triglycerides and a very small amount of glycerophospholipids showed a slight increase. (*p* < 0.01). The total lipid content fell back to the level of the control group. This indicates that stimulation by palmitic acid may significantly enhance the glycerophospholipid metabolism in HepaRG cells, and MGIG may significantly inhibit this activation. 

Palmitic acid, also known as hexadecanoic acid (C16:0), is a saturated higher fatty acid that exists widely in nature. Almost all fats contain varying amounts of C16:0. Therefore, the metabolic flux of C16:0 was analyzed by extracting those glycerides and glycerophospholipids containing C16:0 from the lipidome data. As shown in Figure 6A, after palmitic-acid stimulation, the contents of almost all detected lipids that contained C16:0 were significantly increased in the HepaRG cells (1.8–9.4-fold). However, MGIG treatment caused the content of these upregulated lipids to fall back to normal levels. For cells, palmitic acid administered exogenously is not the only source of intracellular C16:0; the cells themselves can also synthesize 16-carbon fatty acids. In order to eliminate the interference of endogenous C16:0 and track the exact changes of lipid intake, C^13^-isotope-labeled palmitic acid was used to stimulate the HepaRG cells. As shown in Figure 6B, the content of the C^13^-isotope-labeled glycerides and glycerophospholipid containing two molecules of C16:0 increased nearly 7-fold in the lipotoxicity model, but, after intervention with MGIG, they both decreased significantly and even recovered to normal levels. These results show that MGIG can inhibit the cellular uptake of saturated fatty acids (such as C16:0) to synthesize triglycerides and phospholipids, thereby inhibiting the formation of rigid and nonliquid membrane phospholipids containing C16:0 chains. Finally, MGIG can maintain the fluidity of the cell membrane through reducing the saturation of the cell membrane.

**Figure 7:**
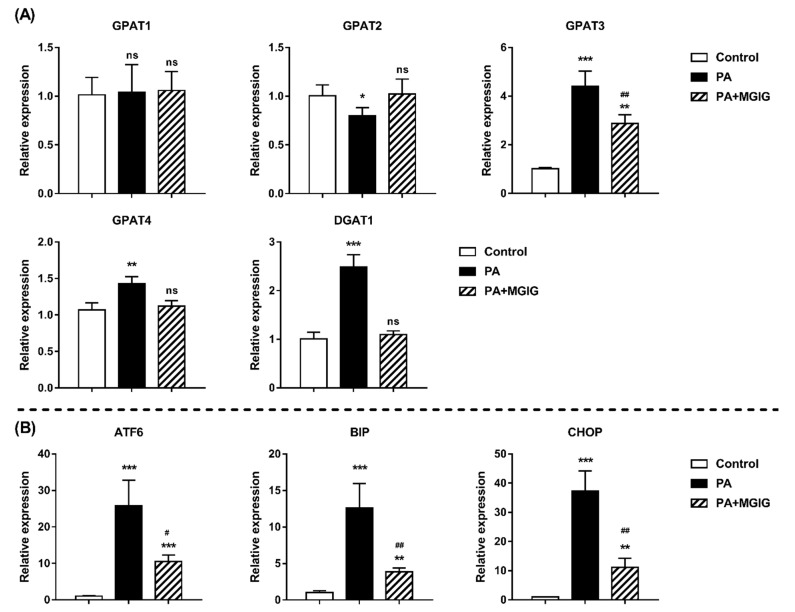
The effect of palmitic acid (PA) and magnesium isoglycyrrhizinate (MGIG) on the gene expression of (**A**) the triglyceride synthesis enzymes GPAT1/2/3/4 and DGAT1 and (**B**) the reticulum stress-related factors ATF6, BIP, and CHOP. Data are expressed as the mean ± SD (*n* = 5). The Control group was untreated cells; the PA group was cells administered 0.2 mM PA for 24 h; and the PA + MGIG group was cells administered 0.2 mM PA plus 0.25 mM MGIG for 24 h. In the statistical analysis between the other groups and the control group, “ns” means *p* > 0.05, * *p* < 0.05, ** *p* < 0.01, and *** *p* < 0.001. In the statistical analysis between the PA group and PA + MGIG group, ^#^ *p* < 0.05 and ^##^ *p* < 0.01. GPAT: Glycerol 3-phosphate acyltransferase; DGAT: Diacylglycerol acyltransferase; ATF6: Activating transcription factor 6; BIP: Binding immunoglobulin protein; CHOP: C/EBP-homologous protein.

Triglycerides (TAGs) are an important form of energy storage for the body. In most mammalian tissues, TAGs are mainly synthesized through the glycerol 3-phosphate pathway. First, glycerol 3-phosphate and two molecules of fatty acyl-CoA are condensed to form phosphatidic acid under the catalysis of glycerol 3-phosphate acyltransferase (GPAT). The phosphatidic acid is then dephosphorylated to form diglycerides (DAGs). DAGs are converted into TAGs by adding another molecule of fatty acyl-CoA under the catalysis of diacylglycerol acyltransferase (DGATs) [27]. All glycerolipids are mainly synthesized in the mitochondrial and endoplasmic reticulum, and GPATs are the rate-limiting enzymes in glycerolipid synthesis [28,29]. There are four subtypes of GPAT, of which GPAT1 and GPAT2 are mainly expressed in the mitochondria and GPAT3 and GPAT4 are mainly expressed in the ER. According to the lipidome data, MGIG had a significant downregulating effect on excessive reserves of TAGs under the lipotoxicity model. Thus, in order to further explore the effect of MGIG on lipids, we investigated the changes in gene expression of related metabolic enzymes (GPAT1/2/3/4 and DGAT1) in the glycerol 3-phosphate pathway (Figure 7). The results showed that after PA stimulation, in addition to GPAT1 and GPAT2, the expression levels of GPAT3, GPAT4, and DAGT1 in the HepaRG cells were all significantly upregulated (1.4–4.4-fold, *p* < 0.01), indicating that the synthesis of TAGs was significantly activated. However, after intervention with MGIG, this upward trend was reversed. The gene expression of GPAT3 that was upregulated in the lipotoxicity model decreased two-fold, and the expression levels of GPAT4 and DAGT1 were even restored to the levels of normal cells. This indicates that MGIG can reduce excessive lipid accumulation in HepaRG cells by inhibiting TAG biosynthesis.

## 3. Discussion and Conclusions

In the case of excessive calorie intake, fat will accumulate for energy storage. Adipocytes are specialized cells that store excess lipids. However, the lipid overloading of adipocytes causes lipids to accumulate in nonadipose tissues, including the pancreas, liver, muscle, and heart, causing cell dysfunction and associated pathologies. This lipotoxicity is a fundamental contributor to various metabolic diseases [30,31]. The endoplasmic reticulum (ER) is the major site for the synthesis of sterols and phospholipids, which constitute most of the lipid components of biological membranes. Many enzymes and regulatory proteins involved in lipid metabolism are located in the ER. Thus, the ER plays a vital role in controlling the membrane lipid composition and membrane lipid homeostasis of all cell types [32]. The stability of the internal environment of the ER is the basic condition under which the function of the ER is realized, so the ER has a powerful dynamic balancing system. However, there are still many factors that may cause an imbalance of ER homeostasis and thus can lead to ER stress (ERS). Studies have shown that palmitic-acid (PA) stimulation can cause the upregulation of many stress responses in cells, including ERS [33]. Our research also proved that the ER membrane of HepaRG cells suffered morphological damage after PA stimulation, including the formation of fragmented ice floe structures. In addition, after PA stimulation, for the rate-limiting enzyme (GPAT) of glyceride synthesis in HepaRG cells, the gene expression of GPAT3 and GPAT4 subtypes in ER increased significantly, while the expression levels of GPAT1 and GPAT2 subtypes in the mitochondria did not change. Researchers have increasingly focused on the correlation between ERS and lipotoxicity. It is likely that relieving ERS can prevent lipotoxicity in several organs, suggesting that ERS might potentially be an untapped therapeutic target for diseases associated with lipid accumulation [34]. Therefore, we further investigated the regulation effect of MGIG on ERS in the lipotoxicity model. Three ERS-related proteins were selected to study for changes in gene expression, including activating transcription factor 6 (ATF6), binding immunoglobulin protein (BIP), and C/EBP-homologous protein (CHOP) [35]. ATF6 is an ER transmembrane ERS sensor and an important regulator in the apoptosis and autophagy pathways caused by ERS. BIP is the main molecular chaperone located in the ER, and is considered to be a marker protein of ERS. CHOP, as a transcription factor, plays an important role in ERS-mediated programmed necrosis. As shown in Figure 7, after PA stimulation, the gene expression levels of ATF6, BIP, and CHOP in the HepaRG cells increased significantly (12.6–37.1-fold). After MGIG intervention, although the gene expression level of these proteins was still higher than normal level, the fold increase was significantly reduced (down 2.5–3.3-fold). It is therefore suggested that MGIG can protect HepaRG cells by alleviating the ERS caused by lipotoxicity after PA stimulation.

In this study, we first used the HepaRG cell model to evaluate the total proliferation rate and DNA synthesis rate, in order to confirm the protective effect of MGIG on liver cell survival. In addition, we confirmed that MGIG also has a positive regulatory effect against the morphological damage of organelles caused by the cell stress response under hepatic lipotoxicity. MGIG can maintain ER membrane fluidity and mitochondrial membrane integrity. Subsequently, we sought to explain this hepatoprotective effect of MGIG from a metabolic viewpoint. Through metabolomics and lipidomics analysis, we obtained information about endogenous metabolites involved in lipid metabolism, cholesterol and hormone metabolism, amino acid metabolism, purine and pyrimidine metabolism, and TCA and carbohydrate metabolism. The results showed that, under PA-induced lipotoxicity, most of the differential metabolites in HepaRG cells were concentrated in lipid metabolism and nucleotide metabolism. The content of the former increased significantly, while the content of the latter decreased significantly. However, MGIG was able to significantly reverse this phenomenon; that is, MGIG had a reversal effect on the metabolic inhibition of nucleotides and the metabolic activation of lipids caused by lipotoxicity.

After further analysis of the lipidomic profile, it was found that glycerides and glycerophospholipids are the two main categories of lipids most affected by PA stimulation. In addition, metabolic flux analysis found that, under the lipotoxicity model, the content of saturated lipids containing C16:0 chains in HepaRG cells increased significantly, but the content decreased significantly and even returned to normal levels following the use of MGIG. In other words, MGIG can reduce membrane saturation by inhibiting the cellular uptake of saturated fatty acids (such as palmitic acid/C16:0) to synthesize membrane phospholipids, thereby maintaining membrane fluidity (such as ER membranes). Moreover, MGIG can reduce the excessive lipid accumulation in HepaRG cells after PA stimulation by downregulating the expression of metabolic enzymes (GPATs and DAGTs) involved in cellular TAG biosynthesis. Finally, the downregulation of ERS after MGIG intervention also confirmed that MGIG is beneficial for hepatocytes under lipotoxicity. However, as a long-term used hepatoprotective agent, MGIG has various protective effects on the liver, and lipotoxicity is also only one of the causes for NASH. Therefore, although we have found that MGIG can antagonize the development of lipotoxicity by interfering with endogenous lipid metabolism, whether this anti-lipotoxic effect will eventually influence the development of NASH requires further research.

## 4. Materials and Methods

### 4.1. Chemicals and Reagents

The test solvent of magnesium isoglycyrrhizinate (MGIG) was a magnesium isoglycyrrhizinate injection provided by Chia Tai TianQing Pharmaceutical Group Co., Ltd. (Nanjing, China). The content of MGIG in the injection was 5 mg/mL Palmitic acid (PA) purchased from Sigma-Aldrich (St. Louis, MO, USA). BSA (fatty acid free) was purchased from Solarbio (Beijing, China). Acetonitrile, methanol, and isopropanol were of LC-MS grade and were purchased from Merck (Darmstadt, Germany). Formic acid, ammonium formate, and methyl tert-butyl ether were purchased from Aladdin (Shanghai, China). Standards of triglycerides (TAG, 17:0-17:1-17:0 D5) and phosphatidylglycerol (PG, 17:0) were obtained from Avanti Polar Lipids (Alabaster, AL, USA). Ultrapure water was prepared using a Milli-Q purification system (Millipore, Bedford, MA, USA) and 5-^13^C-glutamine was purchased from Cambridge Isotope Laboratories, Inc. (Andover, MA, USA). RPMI 1640 medium, fetal bovine serum (FBS), and penicillin–streptomycin (10,000 U/mL) were purchased from Gibco BRL (Grand Island, NY, USA). 

The Cell Counting Kit-8 was purchased from Jiangsu KeyGEN BioTECH Co., Ltd. (Nanjing, China). The BeyoClick™ EdU Cell Proliferation Kit with Alexa Fluor 488, 4% Paraformaldehyde, Triton X-100, Hoechst 33342, ER-Tracker Red, and DAPI staining solution were purchased from Beyotime Biotechnology (Shanghai, China). TMRM was purchased from MedChemExpress (Monmouth Junction, NJ, USA).

### 4.2. Cell Culture

HepaRG cells are terminally differentiated hepatic cells that retain many characteristics of primary human hepatocytes. HepaRG cells were purchased from Thermo Fisher Scientific (Waltham, MA, USA). The cells were cultured in RMPI 1640 medium supplemented with 10% FBS and 1% penicillin–streptomycin at 37 °C, with 5% CO_2_. The medium was changed every two days.

### 4.3. Cell Viability Assay

We used the CCK-8 method to investigate changes in cell viability. HepaRG cells were first seeded into a 96-well plate at a density of 5.0 × 10^3^ cells/well. Subsequently, after incubation at 37 °C in a humidified atmosphere with 5% CO_2_ for 24 h, the cells were treated with single PA or PA plus MGIG at the investigated concentrations for up to 24 h. Six replicates were created for each measurement. Finally, 10 μL of the CCK-8 reagent was added into each well, and the cells continued to incubate at 37 °C for 30 min. The absorbance was measured at 450 nm on a Synergy H1 Hybrid Reader (BioTek Instruments, Inc., Winooski, VT, USA). Cell viability was calculated using the following formula: (tested group absorbance value/untested group absorbance value) × 100%.

### 4.4. Cell Proliferation Assay

The CCK-8 method is a cell proliferation detection method based on cell viability. It can detect the overall proliferation of cells, but not single proliferating cells. By incorporating a thymidine analogue (EdU) during DNA synthesis and then using a click reaction to label the incorporated EdU with Alexa Fluor 488 (a fluorescent probe), single proliferating cells can be detected. HepaRG cells were first seeded into a 12-well plate at a density of 1.0 × 10^5^ cells/well. After incubation at 37 °C with 5% CO_2_ for 24 h, the cells were treated with 0.2 mM PA or 0.2 mM PA plus 0.25 Mm MGIG for 24 h. The drug was then withdrawn, and EDU reagent was added before incubation for 2 h. Subsequently, the EDU was withdrawn, and the fix reagent (4% paraformaldehyde) and permeabilization reagent (Triton X-100) were then added in sequence and incubated for 15 min each. Finally, Alexa Fluor 488 reagent was added to stain the newly synthesized DNA, and Hoechst 33342 reagent was added to stain the nuclei. The cells were observed under a Lionheart™ FX Automated Microscope (BioTek Instruments, Inc., Winooski, VT, USA). 

### 4.5. Cell Damage Assay

HepaRG cells were seeded into a 12-well plate at a density of 1.0 × 10^5^ cells/well. After incubation for 24 h, the cells were then treated with 0.2 mM PA or 0.2 mM PA plus 0.25 Mm MGIG for 24 h. After administration, ER-Tracker Red reagent and TMRM reagent were used to stain the endoplasmic reticulum and mitochondria of living cells to investigate the endoplasmic reticulum morphology and the functional integrity of the mitochondrial inner membrane of the different test groups. The stained cells were directly observed under a fluorescence microscope (Lionheart™ FX Automated Microscope, BioTek Instruments, Inc., Winooski, VT, USA). TMRM is a positively charged rhodamine dye that can be selectively captured by the negatively charged mitochondrial inner membrane, where it emits a strong red fluorescence. Once the channel hole of the mitochondrial membrane is opened, the TMRM is released into the cytoplasm and its fluorescence is significantly reduced. ER-Tracker Red is a red fluorescent probe with cell membrane permeability. It is highly selective for the endoplasmic reticulum and can be used for specific fluorescent staining of the endoplasmic reticulum of living cells.

### 4.6. Gene Expression Profiling and qPCR

Total RNA was extracted from HepaRG cells using a High Pure RNA Isolation Kit (RNAiso Plus, Takara, Japan) and was reverse transcribed using a PrimeScript™ 1st Strand cDNA Synthesis Kit (Takara, Japan), following the manufacturer’s instructions. mRNA expression was assessed by RT-quantitative PCR using a CFX96 real-time detection system (Bio-Rad, Hercules, CA, USA). Three replicates were created for each measurement. The relative quantification was performed using the 2^−ΔΔCt^ method, and values were normalized to the reference gene β-actin. The cycling conditions were as follows: 95 °C for 5 min, followed by 40 cycles at 95 °C for 15 s, 60 °C for 30 s, and 68 °C for 30 s. Melting curve analysis was performed to verify the specificity of the real-time PCR products. The gene-specific primers used in this study are shown in Supplementary Table S1.

### 4.7. Metabolomics Analysis

#### 4.7.1. Metabolomics Sample Preparation

HepaRG cells were seeded into a six-well plate at a density of 2.0 × 10^5^ cells/well. After incubation for 24 h, the cells were then treated with 0.2 mM PA or 0.2 mM PA plus 0.25 mM MGIG for 24 h. Five samples were prepared for each group. After treatment, the drug was removed, and ultrapure water was added after the cells were washed. The cells were lysed by repeated freezing and thawing. A mixed solvent (methanol:acetonitrile:water = 2:2:1, *v/v*/*v*) was used to extract metabolites from the HepaRG cells. The internal standard of 5-^13^C-glutamine was premixed into the mixed solvent at a final concentration of 1.5 μg/mL. After addition of the extraction solution, the samples were first placed at –20 °C for 1 h, and then centrifuged at 13,000 rpm for 15 min at 4 °C. After centrifugation, the supernatant was collected and evaporated to dryness. Quality control (QC) samples were prepared by taking part of the supernatant of each sample and mixing these parts in equal proportions. The QC samples were also evaporated to dryness. The residue was redissolved in another mixed solvent (acetonitrile:water = 1:1, *v/v*). The dissolved sample was centrifuged again to obtain the supernatant for analysis. 

#### 4.7.2. Metabolomics Sample Detection

The LC-Q/TOF-MS determination was performed using the LC-30A system (Shimadzu, Kyoto, Japan) coupled with the TripleTOF^®^ 5600 system (SCIEX, Framingham, MA, USA). The LC-30A system consists of a LC-30A binary pump, a SIL 30 AC autosampler, and a CTO-30AC oven. The XBridge BEH Amide HPLC column (3.5 μm, inner diameter 4.6 mm × 100 mm) (Waters, Milford, MA, USA) was used for chromatographic separation at a column temperature of 40 °C. The mobile phase consisted of solvent A (containing 5% acetonitrile and 5 mmol/L ammonium acetate, adjusted to pH 9 with ammonia water) and solvent B (acetonitrile). A gradient elution at a flow rate of 0.4 ml/min was carried out: 0–3 min (85% B), 3–6 min (85–30% B), 6–15 min (30–2% B), 15–18 min (2% B), 18–19 min (2–85% B), and 19–26 min (85% B). The TripleTOF^®^ 5600 system was equipped with a Turbo V™ ionization source. The MS data were collected by TOF MS scanning and product ion scanning in the range of 50–1000 Da (*m*/*z*) using data-dependent acquisition methods in negative ion mode. The other test conditions were similar to those in the reference paper [24]. Accurate mass calibration was ensured by the calibration delivery system (CDS), and automatic calibration was carried out every three samples. We inserted one QC sample every five samples. The stability of the signal intensity of the QC samples was used to monitor the reproducibility and stability of the analysis during continuous data acquisition. The results showed that in the QC samples, the coefficient of variation (CV) of the peak areas of the chromatographic peaks of annotated metabolites was 21.14 ± 10.10 % (see Supplementary Figure S1A). Data exploration and peak area integration were performed with PeakView and MultiQuant 2.0 from AB SCIEX, respectively.

#### 4.7.3. Metabolomics Data Analysis

All endogenous metabolites were identified by comparing the retention time and mass spectra (both the MS and MS/MS spectra) of the detected compound with a reference database established in our laboratory [25] and other online metabolome databases, including the Human Metabolome Database (http://www.hmdb.ca, accessed on 1 March 2019), MassBank (http://www.massbank.jp, accessed on 1 March 2019), METLIN (http://metlin.scripps.edu, accessed on 1 March 2019), and MS2T (http://prime.psc.riken.jp/lcms/ms2tview, accessed on 1 March 2019). The raw data were the peak area of each compound normalized by the peak area of the internal standard and the protein concentration of each sample. All data were analyzed on the MetaboAnalyst 4.0 website (https://www.metaboanalyst.ca, accessed on 1 May 2019). We selected the sparse partial least squares discriminant analysis (sPLS-DA) mode for multivariate data analysis. The sPLS-DA algorithm can reduce the number of variables (metabolites) to produce robust and easy-to-interpret models. The performance of the sPLS-DA models can be evaluated using cross-validation (CV) with increasing numbers of components created using the specified number of variables. For our model, we chose to use five-fold CV to evaluate the model performance. The results showed that the error rate of our model was the lowest under the current model settings with five components (see Supplementary Figure S2). This means that the model we build is reliable and credible. In the sPLS-DA score plot, each dot represents the summarized information of all endogenous metabolites measured in each sample. The distance between the dots represents the similarity in the metabolic composition between the samples, i.e., the closer they are clustered together the more similar they were. Progenesis QI (Nonlinear Dynamics, Newcastle, UK) was used for peak picking and alignment to screen the discriminant metabolites that showed significant changes between the treated and untreated groups. A one-way ANOVA analysis was used to evaluate the statistical significance of the differences between the three groups, and FDR < 0.05 was considered statistically significant. Statistical analyses were performed using GraphPad Prism 8.0. All results are expressed as the mean ± SD.

### 4.8. Lipidomics Analysis

#### 4.8.1. Lipidomics Sample Preparation

The administration of HepaRG cells in the lipidomics analysis was the same as that in the metabolomics assay. After the treatment, the cells were washed twice with PBS and then ultrapure water was added to repeatedly freeze and thaw the cells. After the cells were lysed, methanol containing internal standards (TAG and PG) was added to precipitate the proteins. Subsequently, methyl tert-butyl ether was added to the sample solution and shaken for 1 h at room temperature. Finally, ultrapure water was added into the mixture solution and shaken for 15 min. Samples were then centrifuged at 12,000 rpm for 10 min at 4 °C, and the supernatant was evaporated to dryness. QC samples were prepared by taking a portion of the supernatant of each sample and mixing these in equal proportions. The QC samples were also evaporated to dryness. The residue was redissolved in a mixed solvent (acetonitrile:isopropanol:water = 65:30:5, *v/v*). The dissolved sample was centrifuged to obtain the supernatant for analysis. 

#### 4.8.2. Lipidomics Sample Detection

The LC-Q/TOF-MS determination was performed using the Agilent Infinity 1290 II system coupled with the Agilent 6545 Q-TOF system (Agilent, Santa Clara, CA, USA). Chromatographic separation was achieved on a Waters ACQUITY UPLC CSH C18 column (I.D. 2.1 mm × 100 mm, 1.7 μm, Milford, MA, USA) with a gradient elution. The column temperature was set to 65 °C. The flow rate was 0.6 mL/min. The mass spectrometer was equipped with a Dual AJS ESI (electrospray ionization) source. The sample analysis was carried out in the positive ionization mode and the negative ionization mode. When using the positive ionization detection mode, the mobile phase of the liquid system consisted of solvent A (acetonitrile:water = 60:40, *v/v*, containing 10 mmol/L ammonium formate and 0.1% formic acid) and solvent B (isopropanol:acetonitrile = 90:10 *v/v*, containing 10 mmol/L ammonium formate and 0.1% formic acid). The gradient elution was set as follows: 0–1.5 min (15% B), 1.5–2 min (15–30% B), 2–2.5 min (30–48% B), 2.5–11 min (48–82% B), 11–11.5 min (82–99% B), 11.5–12 min (99% B), 12–12.1 min (99–15% B), and 12.1–15.0 min (15% B). When using the negative ionization detection mode, the mobile phase of the liquid system consisted of solvent C (acetonitrile:water = 60:40, *v/v*, containing 10 mmol/L ammonium formate) and solvent D (isopropanol:acetonitrile = 90:10 *v/v*, containing10 mmol/L ammonium formate). The gradient elution was set as follows: 0–2.0 min (15–30% D), 2–2.5 min (30–48% D), 2.5–9.5 min (48–76% D), 9.5–9.6 min (76–99% D), 9.6–10.5 (99% D), 10.5–10.6 min (99–15% D), and 10.6–13.5 min (15% D). The iterative MS/MS mode was used for mass determination. The ESI source conditions were set as follows: TOF MS scan, m/z 60–1700 Da; MS/MS scan, *m*/*z* 60–1700 Da; drying gas temperature, 325 °C; drying gas flow, 8 L/min; nebulizer gas, 35 psi; sheath gas temperature, 350 °C; sheath gas flow, 11 L/min; capillary voltage, 3.5 Kv; capillary outlet voltage, 120 V; nozzle voltage, 1 Kv; cone voltage, 65 V; and collision energy, 25 eV. We inserted one QC sample every five samples to monitor the analysis stability and reproducibility through the stability of the signal intensity of the QC samples. The results showed that in the positive ionization detection mode and the negative ionization detection mode, in the QC samples, the CVs of the peak areas of the chromatographic peaks of annotated lipids were 17.94 ± 12.72 % and 25.99 ± 11.30 %, respectively (see Supplementary Figure S1B).

#### 4.8.3. Lipidomics Data Analysis

The endogenous lipid metabolites were annotated using the MS-DIAL 3.98 software. MS-DIAL is open-source software that was launched as a universal program for untargeted metabolomics. Raw Agilent data files were first converted to ABF format (.abf) using the Reifycs Abf (Analysis Base File) Converter. For annotation of lipids, all lipid categories listed in the MS-DIAL 3.98 software were considered target lipids. The adduct ions were set to [M + H]^+^, [M + NH_4_]^+^, [M + Na]^+^ and [M-H]^−^, and [M + CH_3_COO]^−^. The parameters of peak detection in MS-DIAL were as follows: retention time (0–15 min), mass range (0–1500 Da), MS1 tolerance (0.01 Da), MS2 tolerance (0.025 Da), retention time tolerance (0.15 min), minimum peak height (500 amplitude), minimum mass width (0.05 Da), and minimum score (80%). Using the MS-DIAL program, we first tried to find a lipid metabolite candidate by means of the accurate mass, isotope ratio, and MS/MS similarity. The metabolites which obtained the ‘highest score’ among the metabolite candidates were annotated. This score was the total score from the accurate mass similarity, isotope ratio similarity, and MS/MS similarity. As shown in Supplementary Figure S3, by comparing the accurate mass and mass abundance of the detected mass spectrum with the reference mass spectrum in the MS-DIAL database, some annotated lipids can only describe the length and unsaturation of the carbon chain, such as the lipid of TAG 60:2, while some annotated lipids can accurately describe the specific composition of the carbon chain, such as the lipid of TAG 60:2 (24:0/18:1/18:1). We finally annotated more than 2000 different lipid metabolites under both the positive ion mode and negative ion mode. Then, the peak area integration of each annotated lipid was carried out with MultiQuant 2.0 from AB SCIEX, and the peak area was used to represent the abundance of the target lipid in the sample. A one-way ANOVA analysis was used to evaluate the statistical significance of the differences among the three groups, and *p* < 0.05 was considered statistically significant. Statistical analyses were performed using GraphPad Prism 8.0. All the results are expressed as the mean ± SD.

### 4.9. Cytoscape Analysis

Cytoscape is an open-source software platform that realizes the visualization of complex networks by integrating any type of attribute data. In a Cytoscape plot, each dot represents an identified endogenous metabolite in the samples. The size of the dot represents the average abundance of the investigated endogenous metabolite in the samples. The color of the dot can have different meanings for different purposes. As shown in Figure 4B,C, all endogenous metabolites identified in the samples were visualized using Cytoscape. In these plots, the differential metabolites with significant differences in abundance between two test groups are highlighted by colors. The upregulated metabolites and downregulated metabolites are represented by red dots and blue dots, respectively. The larger the diameter of the dot, the greater the difference between groups. The metabolites with no significant difference between groups are represented by yellow dots. Moreover, as shown in Figure 5B–D, one specific metabolic pathway (the glycerophospholipid pathway) was visualized using Cytoscape. In these visual plots, the color of the dots represents whether a specific target lipid in this pathway was detected in the sample. A black dot means it was not found in the sample, and a blue dot means it was found. The size of the blue dot represents the average abundance of the target lipid in the samples. Assuming that the content of each lipid in the control group is the same, the diameter of all dots is 0.5 cm. The larger the point, the higher the target lipid content in the sample.

## Data Availability

The data presented in this study are available in insert article and supplementary material. The raw data are available on request from the corresponding author.

## Supplementary Materials

Supplementary materials are available online at https://www.mdpi.com/article/10.3390/ijms22115884/s1.

## References

[1] Henne W.M., Reese M.L., Goodman J.M. (2018). The assembly of lipid droplets and their roles in challenged cells. EMBO J..

[2] Brookheart R.T., Michel C.I., Schaffer J.E. (2009). As a Matter of Fat. Cell Metab..

[3] Alves-Bezerra M., Cohen D.E. (2017). Triglyceride Metabolism in the Liver. Compr. Physiol..

[4] Van Herpen N.A., Schrauwen-Hinderling V.B. (2008). Lipid accumulation in non-adipose tissue and lipotoxicity. Physiol. Behav..

[5] Matsuki H., Goto M., Tamai N. (2019). Membrane States of Saturated Glycerophospholipids: A Thermodynamic Study of Bilayer Phase Transitions. Chem. Pharm. Bull..

[6] Lara-Castro C., Garvey W.T. (2008). Intracellular Lipid Accumulation in Liver and Muscle and the Insulin Resistance Syndrome. Endocrinol. Metab. Clin. N. Am..

[7] Yazıcı D., Sezer H. (2017). Insulin Resistance, Obesity and Lipotoxicity. Adv. Exp. Med. Biol..

[8] Rada P., Gonzalez-Rodriguez A., Garcia-Monzon C., Valverde A.M. (2020). Understanding lipotoxicity in NAFLD pathogenesis: Is CD36 a key driver?. Cell Death Dis..

[9] Mota M., Banini B.A., Cazanave S.C., Sanyal A.J. (2016). Molecular mechanisms of lipotoxicity and glucotoxicity in nonalcoholic fatty liver disease. Metabolism.

[10] Neuschwander-Tetri B.A. (2017). Non-alcoholic fatty liver disease. BMC Med..

[11] Basaranoglu M., Ormeci N. (2014). Nonalcoholic fatty liver disease: Diagnosis, pathogenesis, and management. Turk. J. Gastroenterol..

[12] Sui M., Jiang X., Chen J., Yang H., Zhu Y. (2018). Magnesium isoglycyrrhizinate ameliorates liver fibrosis and hepatic stellate cell activation by regulating ferroptosis signaling pathway. Biomed. Pharmacother..

[13] Yang Y.Z., Liu Z.H., Wang S.C., Zhang X.Q., Xu H.J., Yang L., Kong L.D. (2020). Magnesium isoglycyrrhizinate alleviates fructose-induced liver oxidative stress and inflammatory injury through suppressing NOXs. Eur. J. Pharmacol..

[14] Zou X., Wang Y., Peng C., Wang B., Niu Z., Li Z., Niu J. (2018). Magnesium isoglycyrrhizinate has hepatoprotective effects in an oxaliplatininduced model of liver injury. Int. J. Mol. Med..

[15] Wang Y., Wang Z., Gao M., Zhong H., Chen C., Yao Y., Zhang Z., Zhang X., Li F., Zhang J. (2019). Efficacy and safety of magnesium isoglycyrrhizinate injection in patients with acute drug-induced liver injury: A phase II trial. Liver Int..

[16] Cheng Y., Zhang J., Shang J., Zhang L. (2009). Prevention of Free Fatty Acid-Induced Hepatic Lipotoxicity in HepG2 Cells by Magnesium Isoglycyrrhizinate in vitro. Pharmacology.

[17] Xu Q., Wang J., Chen F., Lin K., Zhu M., Chen L., Zhou X., Li C., Zhu H. (2016). Protective role of magnesium isoglycyrrhizinate in non-alcoholic fatty liver disease and the associated molecular mechanisms. Int. J. Mol. Med..

[18] Gjorgjieva M., Sobolewski C., Dolicka D., de Sousa M.C., Foti M. (2019). miRNAs and NAFLD: From pathophysiology to therapy. Gut.

[19] Li B., He X., Jia W., Li H. (2017). Novel Applications of Metabolomics in Personalized Medicine: A Mini-Review. Molecules.

[20] German J.B., Hammock B.D., Watkins S.M. (2005). Metabolomics: Building on a century of biochemistry to guide human health. Metabolomics.

[21] Rattray N.J.W., Deziel N.C., Wallach J.D., Khan S.A., Vasiliou V., Ioannidis J.P.A., Johnson C.H. (2018). Beyond genomics: Understanding exposotypes through metabolomics. Hum. Genom..

[22] Wenk M.R. (2010). Lipidomics: New Tools and Applications. Cell.

[23] Brügger B. (2014). Lipidomics: Analysis of the Lipid Composition of Cells and Subcellular Organelles by Electrospray Ionization Mass Spectrometry. Annu. Rev. Biochem..

[24] Engin A.B. (2017). What Is Lipotoxicity?. Adv. Exp. Med. Biol..

[25] Pereira P.D., Serra-Caetano A., Cabrita M., Bekman E., Braga J., Rino J., Santus R., Filipe P.L., Sousa A.E., Ferreira J.A. (2017). Quantification of cell cycle kinetics by EdU (5-ethynyl-2′-deoxyuridine)-coupled-fluorescence-intensity analysis. Oncotarget.

[26] Shen Y., Zhao Z., Zhang L., Shi L., Shahriar S., Chan R.B., Di Paolo G., Min W. (2017). Metabolic activity induces membrane phase separation in endoplasmic reticulum. Proc. Natl. Acad. Sci. USA.

[27] Nagle C.A., Vergnes L., Dejong H., Wang S., Lewin T.M., Reue K., Coleman R.A. (2008). Identification of a novel sn-glycerol-3-phosphate acyltransferase isoform, GPAT4, as the enzyme deficient in Agpat6−/− mice. J. Lipid Res..

[28] Beigneux A.P., Vergnes L., Qiao X., Quatela S., Davis R., Watkins S.M., Coleman R.A., Walzem R.L., Philips M., Reue K. (2006). Agpat6--a novel lipid biosynthetic gene required for triacylglycerol production in mammary epithelium. J. Lipid Res..

[29] Chen Y.Q., Kuo M.S., Li S., Bui H.H., Peake D.A., Sanders P.E., Thibodeaux S.J., Chu S., Qian Y.W., Zhao Y. (2008). AGPAT6 is a novel microsomal glycerol-3-phosphate acyltransferase. J. Biol. Chem..

[30] Unger R.H., Scherer P.E. (2010). Gluttony, sloth and the metabolic syndrome: A roadmap to lipotoxicity. Trends Endocrinol. Metab..

[31] Wang S., Kaufman R.J. (2014). How does protein misfolding in the endoplasmic reticulum affect lipid metabolism in the liver?. Curr. Opin. Lipidol..

[32] Fagone P., Jackowski S. (2009). Membrane phospholipid synthesis and endoplasmic reticulum function. J. Lipid Res..

[33] Sargsyan E., Artemenko K., Manukyan L., Bergquist J., Bergsten P. (2016). Oleate protects beta-cells from the toxic effect of palmitate by activating pro-survival pathways of the ER stress response. Biochim. Biophys. Acta BBA Mol. Cell Biol. Lipids.

[34] Han J., Kaufman R.J. (2016). The role of ER stress in lipid metabolism and lipotoxicity. J. Lipid Res..

[35] Oyadomari S., Mori M. (2004). Roles of CHOP/GADD153 in endoplasmic reticulum stress. Cell Death Differ..

