# Supplementary material for: Magnesium Isoglycyrrhizinate Reduces Hepatic Lipotoxicity through Regulating Metabolic Abnormalities
Source: Int J Mol Sci. 2021 May 30;22(11):5884. doi: 10.3390/ijms22115884 (PMC8198484; doi:10.3390/ijms22115884)
Supplement: Supplementary file 1 [file ijms-22-05884-s001.zip › ijms-1177392-supplementary.pdf]

Supplementary Table S1 Sequences of specific primers for the genes studied in this study.

Gene	*5′-3′ Sequence*	*3′-5′ Sequence*
β-actin	CATGTACGTTGCTATCCAGGC	CTCCTTAATGTCACGCACGAT
DGAT1	CAATCTGACCTACCGCGATCT	TCGATGATGCGTGAGTAGTCC
GPAT1	GAGATGTGCATAAGGGCATGT	AATTGCCTCTTGTACTCTACTGC
GPAT2	TGCACCGTGTGACATAGACC	GCACTGACGATGTATTCCTGC
GPAT3	ACTTTAGAGTGGGCCACAATACG	TGGGTGACTCATCTCTTTGGAT
GPAT4	GGTATCCGCAAACTCTACATGAA	CCACTTCGACGAATCTCTTTGA
ATF	CCTCTGCGCTGGAATCAGTC	TTCTTTCTCGTCGCCTCTTTTT
BIP	GACATCAAGTTCTTGCCGTTCA	CTTTGTTTGCCCACCTCCAATA
CHOP	AGAACCAGGAAACGGAAACAGA	TCTCCTTCATGCGCTGCTTT

Supplementary Fig.1

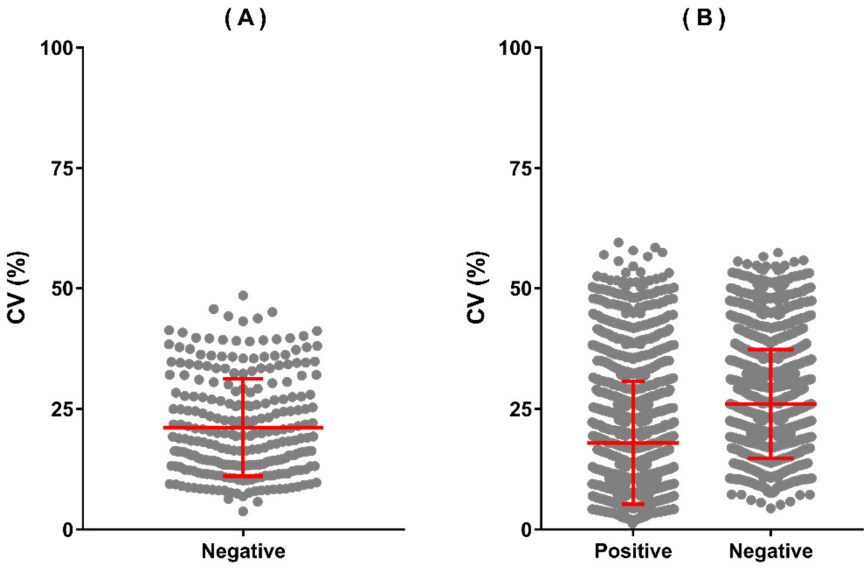

Supplementary Fig.1 The coefficient of variation (CV) of the peak areas of the chromatographic peaks of the annotated metabolites or lipids in the quality control (QC) samples during metabolomics analysis (A) or lipidomics analysis (B). Data are expressed as the mean ± SD (n=5). For metabolomics analysis, the samples were detected only in the negative ionization mode. For lipidomics analysis, the samples were detected in the positive ionization mode and in the negative ionization mode, respectively.

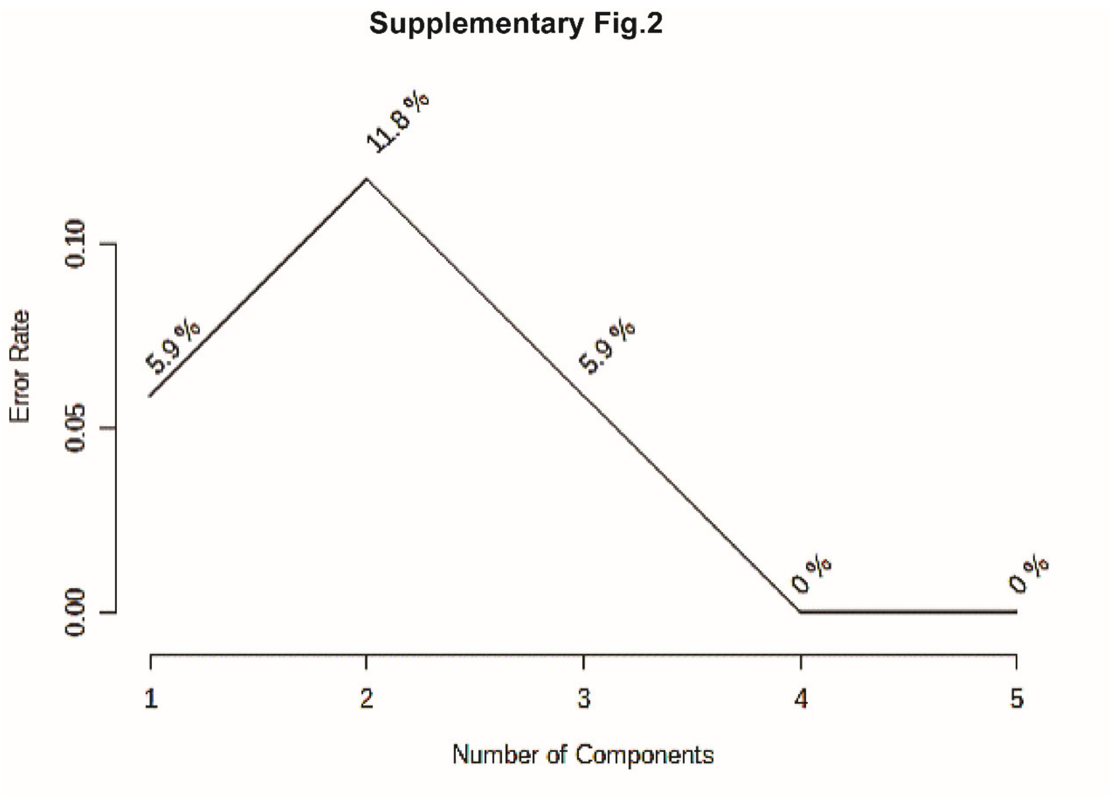

Supplementary Fig.2 The plot of Sparse PLS-DA Classification Error Rates using 5-fold cross-validation.

Supplementary Fig.3

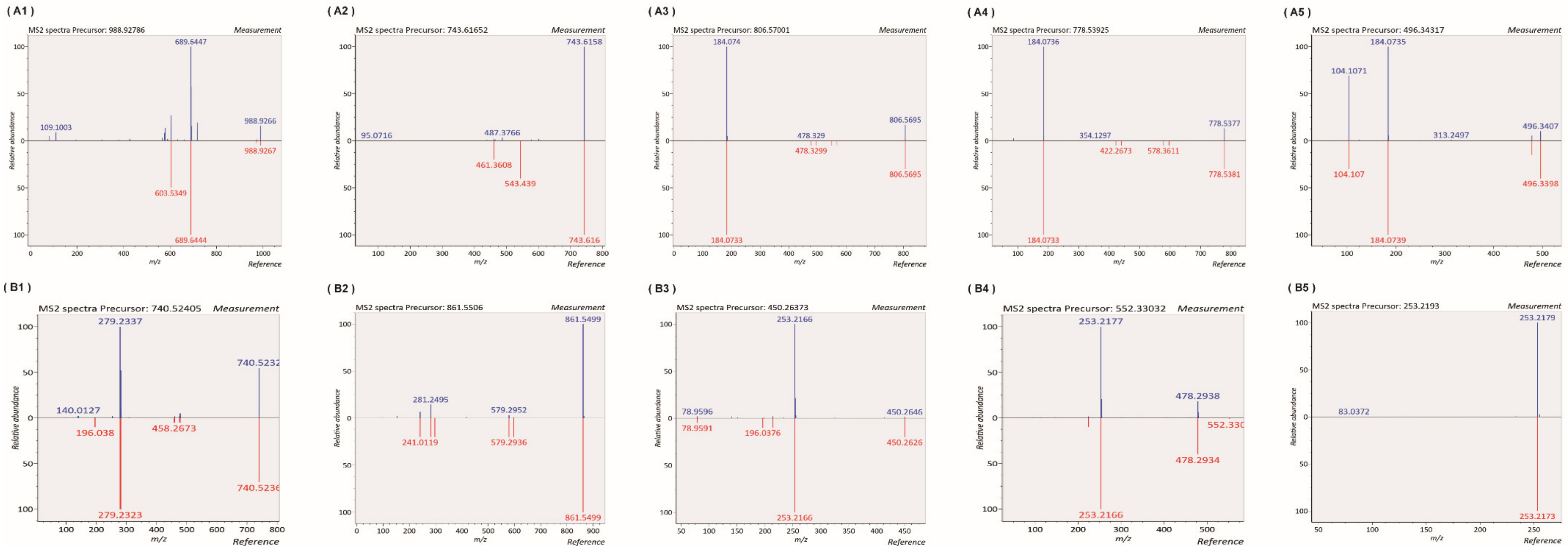

Supplementary Fig.3 Typical MS/MS mass spectrums for lipid annotation in lipidomics analysis under positive ionization detection mode (A) and negative ionization detection mode (B). In each MS/MS mass spectrum listed, blue is the real mass information measured in the sample, and red is the reference mass information of annotated lipids retrieved by the MS-DIAL program. These annotated lipids listed are A1 : TAG 60:2 (24:0/18:1/18:1), A2 : TAG 42:1 (12:0/12:0/18:1), A3 : PC 38:6, A4 : PC 36:6 (12:0/24:6), A5 : LPC 16:0, B1: PE 36:2 (18:1/18:1), B2: PI 36:2 (18:1/18:1), B3: LPE 16:1, B4: LPC 16:1, B5: FA 16:1.

Supplementary Fig.4

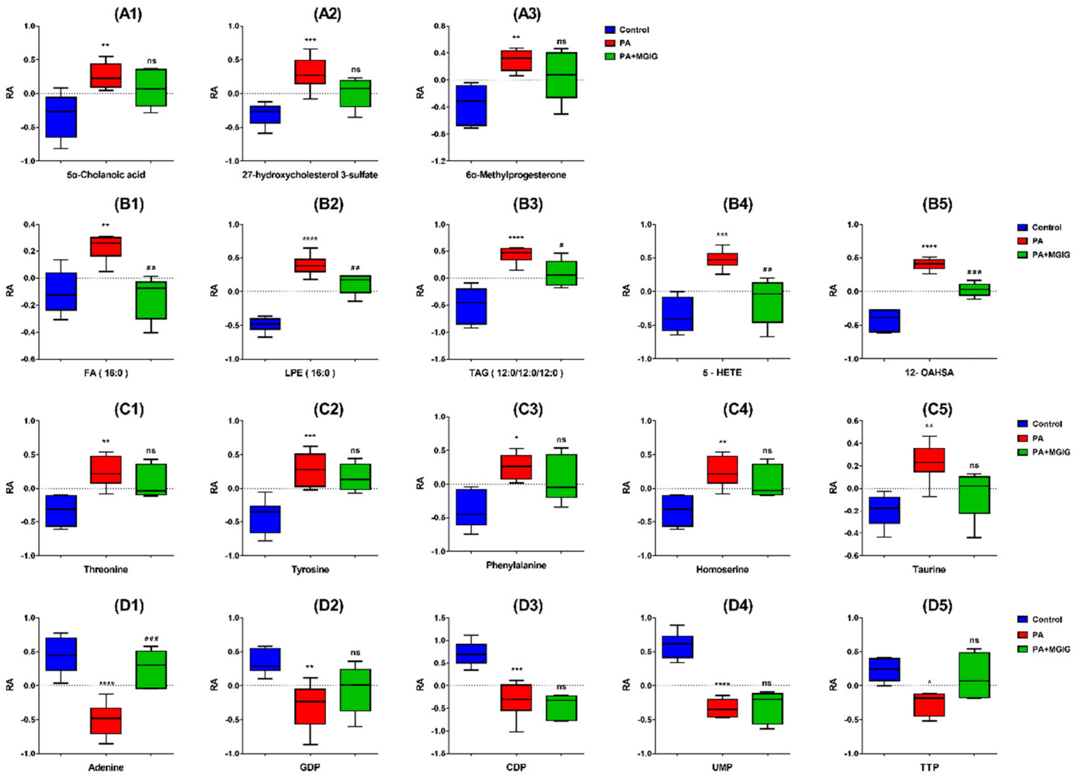

Supplementary Fig.4 Relative abundance (RA) of typical discriminant metabolites in HepaRG cells involved in the metabolic pathways of (A) cholesterol metabolism, (B) lipids metabolism, (C) amino-acid metabolism and (D) nucleotide metabolism. Data are expressed as the mean ± SD (n=5). RA is the data normalized by logarithmic transformation and Pareto scaling. The Control group was untreated cells; the PA group was cells administered 0.2 mM PA for 24 h; and the PA + MGIG group was cells administered 0.2 mM PA plus 0.25 mM MGIG for 24 h. In the statistical analysis carried out between the PA group and Control group, *$p < 0.05$; **$p < 0.01$, ***$p < 0.001$; ****$p < 0.0001$. In the statistical analysis carried out between the PA+MGIG group and PA group, "ns" means $p > 0.05$, $^{\#}$ $p < 0.05$, $^{\#\#}$ $p < 0.01$, $^{\#\#\#}$ $p < 0.001$.
